# User Preferences for an Image-Assisted Dietary Recall: Qualitative Study Comparing 3 Dietary Assessment Methods

**DOI:** 10.2196/79565

**Published:** 2025-12-30

**Authors:** Janelle D Healy, Christina M Pollard, Clare E Collins, Barbara A Mullan, Megan E Rollo, Satvinder S Dhaliwal, Richard Norman, Sharon I Kirkpatrick, Tracy A McCaffrey, Clare Whitton, Amira Hassan, Fengqing Zhu, Deborah A Kerr

**Affiliations:** 1 Curtin School of Population Health Curtin University Perth, Western Australia Australia; 2 Enable Institute Curtin University Perth, Western Australia Australia; 3 School of Health Sciences, College of Health, Medicine and Wellbeing University of Newcastle Australia Newcastle, New South Wales Australia; 4 Food and Nutrition Research Program, Hunter Medical Research Institute University of Newcastle Australia Newcastle, New South Wales Australia; 5 Health & Social Sciences Singapore Institute of Technology Singapore Singapore; 6 Office of the Provost Singapore University of Social Sciences Singapore Singapore; 7 Obstetrics & Gynaecology Academic Clinical Program Duke NUS Medical School National University of Singapore Singapore Singapore; 8 School of Public Health Sciences University of Waterloo Waterloo, ON Canada; 9 Department of Nutrition, Dietetics and Food Monash University Melbourne, Victoria Australia; 10 School of Medical and Health Sciences Edith Cowan University Joondalup, Western Australia Australia; 11 Curtin Medical Research Institute Curtin University Perth, Western Australia Australia; 12 School of Electrical and Computer Engineering Purdue University West Lafayette West Lafayette, IN United States

**Keywords:** 24-hour recall, qualitative research, interview, Automated Self-Administered Dietary Assessment Tool, Intake24, mobile food record, image-assisted dietary assessment, controlled feeding, acceptability, mobile technology

## Abstract

**Background:**

Technology-assisted 24-hour dietary recall (24HR) methods offer the potential for scalable population dietary assessment, but current challenges include balancing accuracy and cost against participant burden and acceptability of these methods. Qualitative methods present a novel approach to understanding potential barriers and enablers to the acceptability of 24HR methods, but remain relatively unexplored.

**Objective:**

This study aimed to explore users’ experience, acceptability, and preferences for 3 technology-assisted 24HR methods.

**Methods:**

Participants in a crossover controlled feeding study were invited to undertake a poststudy interview. Initially, the feeding study participants were randomized into one of three separate feeding days where they consumed breakfast, lunch, and dinner on a single day. On the following day, they undertook a 24HR via the Automated Self-Administered 24-hour Dietary Assessment Tool (ASA24), Intake24, or an Image-Assisted Interviewer-Administered 24-hour dietary recall (IA-24HR). When assigned to IA-24HR, participants viewed the images they captured with a mobile food record (mFR) app on the feeding day during the interview. On completing all 3 methods, 26 participants (ages 21 to 56 years) undertook semistructured interviews. The interview audio recordings were transcribed, and inductive content analysis was undertaken.

**Results:**

Overall, participants wanted the 24HR methods to be easy, with the technology features of all methods considered helpful. A total of 5 content categories described users’ experiences of the three 24HR methods: (1) “Put my food in the list,” (2) “It’s really hard to know portions,” (3) ASA24 “was a painful process,” (4) access to “images helped jog my memory,” (5) Intake24 is “fairly quick,” and (6) IA-24HR method preference. Participants expressed a preference for taking images with the mFR app. IA-24HR helped participants recall food and beverages consumed and increased perceptions of recall accuracy.

**Conclusions:**

This novel qualitative research found that 24HR methods need to be as easy as possible for users. The participant burden of food and beverage identification and portion size estimation was evident across methods. Findings highlight the importance of using qualitative methods to explore user preferences for dietary assessment methods and confirm the need to reduce the user burden associated with 24HR methods. People want embedded technologies to enhance digitized versions of the traditional 24HR methods. The use of their own food images within the mFR app is an example of digital advancements within scalable 24-hour dietary assessments.

**Trial Registration:**

Australia New Zealand Clinical Trials Registry ACTRN12621000209897; www.anzctr.org.au/Trial/Registration/TrialReview.aspx?id=381165

**International Registered Report Identifier (IRRID):**

RR2-10.2196/32891

## Introduction

Population-level dietary advice and policy are informed by data from accurate and reliable dietary assessment methods, with the 24-hour dietary recall (24HR) the most common method used to assess usual intakes in population surveillance [1]. 24HR methods balance accuracy and cost of reporting against respondent burden [2,3]. Traditionally, the 24HR was conducted via a structured interview to capture detailed information about food and beverages consumed in the previous 24 hours. Increasingly, self-administration of the structured interview via prompts within a web-based tool has been primarily driven by the need to reduce administration costs [2]. The Automated Multiple-Pass Method (AMPM) consists of 5 steps that guide the interviewer through the recall process using standardized questions and probes [4]. An example of a widely used approach that can be self-administered is the online Automated Self-Administered 24-Hour Dietary Assessment Tool (ASA24) [5] developed with automated prompts and checks to maintain consistency with the AMPM protocol. A second online example is Intake24, developed through iterative processes starting with the Self-Completed Recall and Analysis of Nutrition (SCRAN24) for children, using think-aloud, eye tracking, and semistructured interviews with adults and children, resulting in fewer prompts for food items and portions to enhance the user experience [6-9]. A feature of these online methods is the use of static food images with a range of portion size choices to guide portion size estimation by the respondent [10]. Both online 24HR methods have been adapted for use in several countries and evaluated for accuracy [7,11,12]. Evaluation of developing methods is often based on nutrient accuracy against an established dietary assessment method. For example, Ocke et al [13] compared an app-based diet record to an established 24-hour recall for accuracy, but not the user experience. Alternatively, the developers may assess user acceptability of a dietary assessment method in isolation [14]. There is little published evidence of qualitative investigations of comparative user acceptability of an image-assisted dietary assessment method against two established online methods.

Customarily, 24HRs are unannounced to reduce the likelihood of reactivity, where respondents change their behavior due to awareness that their food intake is being assessed [15]. Endeavors to enhance the user experience while balancing the need for accuracy have led researchers to explore image-assisted approaches to supplement the 24HR [10]. These dynamic images and videos collected by the respondent at the time of eating are then available to assist with the recall process on the following day [10,16,17]. However, there is concern that capturing food images at the time of recording may lead to reactivity but may improve the documentation of food and beverages consumed [16,17]. It remains unknown how this method compares for accuracy and acceptability with 24HR computer applications using static images as with ASA24 and Intake24. In a controlled feeding study, Accuracy and Cost-Effectiveness of Technology-assisted Dietary Assessment (ACE-TADA) [12,18], where food and beverages are known, we compared the accuracy of recalls completed using ASA24, Intake24, and an Image-Assisted interviewer-administered 24HR (IA-24HR), where during the interview process participants viewed images they captured with the mobile Food Record (mFR) app [19]. A research dietitian, a trained analyst, estimated the portion size from the images. The IA-24HR method has the potential to reduce respondent burden, but comparative user acceptability of this method is yet to be fully evaluated [10].

While the acceptability of 24HR for users is widely acknowledged, there are surprisingly few qualitative studies designed to explore the comparative user experience of 24HR methods. Qualitative studies provide valuable insights into the user experience, thus improving the acceptability of 24HR. Researchers in New Zealand [14] investigated the user acceptability of Intake24 in the NZ population, but did not compare the experience to other 24HR methods. Moyen et al [20] found Canadian participants appreciated an image-based food record compared to ASA24 when assessed quantitatively from a system usability scale in a 2-arm crossover study. A qualitative study of 55 Australian university students (75% women) used a think-aloud method to identify perceived problems in two self-administered 24HRs [21]. There was a strong preference for Intake24 over ASA24, with participants reporting that the recall cues felt less repetitive for Intake24. We were unable to source any qualitative investigations that compared ASA24, Intake 24, and an image-based food recall method for user acceptability from the participants’ perspective.

Understanding the user experiences and perceptions of the IA-24HR method compared with others, such as ASA24 [5] and Intake24 [6,8], can inform methodological decisions. The IA-24HR method has the potential to reduce respondent burden for 24HRs, but user comparative acceptability and impact on reactivity are yet to be fully evaluated [10]. No studies to date have been conducted on in-depth interviews with users of the three 24HR technology-assisted methods. This study aimed to compare the relative user perception and acceptability of 3 technology-assisted 24HR methods from the perspective of the users’ experience, using qualitative methods.

## Methods

### Study Design

Eligible participants completed a baseline demographic survey before attending a research facility to consume 3 meals (breakfast, lunch, and dinner) on each of the 3 feeding days, a week apart. On each feeding day, participants selected from a menu for each meal and consumed meals ad libitum. The menu offered a variety of hot and cold meal options for the 3 meals, to be prepared in the study kitchen. A sample version of the 3 rotating menus (Multimedia Appendix 1). Participants were able to leave the facility between meals, and there were no restrictions on the consumption of food and beverages between meals. Participants then undertook the 24HR recall the following day. When assigned to the IA24, they accessed their images during the recall process [12]. Each technology-assisted method was used to assess one day of dietary intake for each individual. Multimedia Appendix 2 compares the 3 technology-assisted methods, ASA24 [5], Intake24 [6,22], and IA-24HR [12,18] with Multimedia Appendix 3 showing the screenshots for each method. The nutrient analysis for each method was conducted using the Australian nutrient database, Australian Food and Nutrient Database (AUSNUT 2011-2013) [23]. Participants were randomized to the 24HR method order before their first meal, and each 24HR was completed on the day following each feeding day. ASA24 and Intake24 were self-administered, automated recalls, using multiple passes conducted on participants’ own devices. When assigned to the IA-24HR method, participants took before and after images of their meals within the mFR app installed on their device. These images were automatically uploaded either by WiFi or 4G to the study website. A fiducial marker (standard object of color and size to assist with food identification and portion estimation) was included in each image [24-26]. The images were returned to participants after midnight for labelling of the content of their mFR images before their interview. These labels linked to a food composition database not visible to participants, containing 372 food and beverage items (AUSNUT 2011-13) [23]. The labelled images were available during the IA-24HR via a video call. The interviewer confirmed the contents of the images and recorded participants’ estimations of portion sizes eaten using the Food Model Booklet (Multimedia Appendix 3), which was used in the Australian Health Survey [27].

Following completion of the three 24HR methods within the controlled feeding study, participants were invited to participate in individual semistructured interviews. An interpretivist epistemological approach was adopted by the researcher to understand the subjective meaning that participants attributed to their experience with the different dietary recall methods. This approach acknowledges that acceptability and preference are based on personal experience and context [28], communicated by the words used. A qualitative descriptive study design was used to capture rich understandings of these subjective experiences of the 24HR methods described by the participants and interpretations by the researchers [29].

A total of 35 participants who completed all three 24HR methods were purposefully invited by email to participate in an online semistructured interview. A purposefully selected sample was invited to provide a rich exploration of the comparative 24HR experience [30]. A quota sample from the main study of 50:50 (women:men) and approximately equal numbers from each age group (18-34, 35-49, and 50+ years) was used to increase the potential for diverse food and technology experiences [21]. A total of 7 participants did not respond, and 2 did not attend their interview. Recruitment continued until no new points were stated in the interviews [31]. The semistructured interviews (n=26) were conducted by JH. Participants were aware that JH was a researcher on the feeding study. A digital voucher (AUD $50; US $33) was provided in recognition of the time taken for the interview. Digital informed consent and verbal confirmation were obtained before audio recording.

The interview script and prompts were developed by the research team (JDH, CMP, and DAK), experienced in dietary assessment and qualitative methods (see Multimedia Appendix 4 for the interview script). Interviews were conducted online and included an introduction to explain the research, highlighting that participants’ opinions in their own words were sought and that there were no right or wrong answers. The interviewer (JDH) briefly described the three 24HR methods using screenshots of the platform home pages to help participants differentiate between them. The interview questions asked about participants’ experience using each method, in the order they completed the 24HR. Questions aimed to elicit an overall opinion of each method and the features of the 24HR method that were helpful or challenging. The final question asked participants if they would undertake a 24-hour fast again, which method they would prefer, and why. Interviews employed open-ended questions with probing to encourage participants to elaborate on their experiences and perspectives, while acknowledging the researcher’s active role in the interpretive process [29].

Audio recordings identified by a 4-digit code were professionally transcribed, and participants were invited to review them for accuracy. A total of 5 of the 26 participants reviewed their transcripts, with 2 making minor edits. Transcripts were managed using NVivo software (QSR International). A total of 3 female coders with diverse backgrounds and research experience independently reviewed the transcripts. The coders were guided by inductive content analysis steps to code the transcripts [32] with review by an experienced qualitative researcher (CMP).

### Participants and Recruitment

This study was part of the ACE-TADA study, a crossover controlled feeding study to compare the accuracy of energy and nutrient intake estimation of 4 technology-assisted dietary assessment methods relative to true intake across breakfast, lunch, and dinner. Details of the study protocol and main outcomes have been published previously [12,18]. Briefly, adult participants aged 18-76 years were recruited from Curtin University staff and students in Perth, Australia, by website and email advertisement. Exclusion criteria included those with serious illnesses or medical conditions requiring specialized nutritional interventions (eg, diabetes requiring insulin therapy, cancer treatment in the last 5 years, gastrointestinal disorders, and kidney, heart, liver, or thyroid disease), pregnancy, or dietary restrictions (eg, to lose weight, food allergies or intolerances).

### User Experience Analysis

Inductive content analysis was used to examine how participants constructed meaning from their experiences with the technology-assisted 24HR methods. Analysis began with two coders extracting quotes from the transcripts that represented participants’ experiences of using each dietary recall method [32]. These quotes were examined to develop inductive codes capturing how participants interpreted and understood their interactions with the different methods. Codes were refined through iterative review processes conducted via discussion among coders, allowing for consideration of diverse perspectives and assumptions. This collaborative approach enabled the development of final categories that summarized participant experiences.

The reporting of these results was guided by the Consolidated Criteria for Reporting Qualitative Research (COREQ) [33] with the checklist in Multimedia Appendix 5. Quotations are italicized with the gender (W=women or M=men) and age group (18-35 years, 36-49 years, and 50+ years) identified, and the 24HR method (ASA24, Intake24, and IA-24HR) when specified.

### Ethical Considerations

Ethical approval was attained from Curtin University Human Research Ethics Office (approval HRE2019-0222) and reciprocal ethics approval from the Department of Health WA Human Research Ethics Committee (Approval 201909.06). The study was registered with the Australian New Zealand Clinical Trials Registry (ACTRN12621000209897). All research design, practices, and reporting of studies conducted in Australia will be aligned with the Australian Code for the Responsible Conduct of Research. A digital voucher of AUD $50 (US $33) was provided in recognition of the time taken for the interview. Digital informed consent and verbal confirmation were obtained before audio recording.

## Results

### Participants

Interviews averaged 31 minutes, with a range of 23 to 43 minutes. A total of 13 of the 26 interviewees were women (n=13), the mean age was 40.5 (SD 9.2 years; <35 years, n=8; 35 to 49 years, n=11; > 50 years, n=7). A total of 14 participants identified as White and 11 as Asian. In total, 25 (96%) participants had attained a bachelor’s degree or higher, and 21 were employed full-time (Multimedia Appendix 6). The randomized order in which participants undertook each 24HR method was evenly distributed. The 24HR method order had been randomized at the first meal on the first feeding day for each participant, and the method, rather than the order in which they were completed, was found to be associated with accuracy [12].

### User Experience

A total of 6 main content categories described the user experience of 24HRs, with quotations used as content category headings: (1) “Put my food in the list” (M5, 36-49 years), (2) “It’s really hard to know portions” (M3, 50+ years); (3) “Using ASA24 is painful”(W3, 50+ years); (4) My mFR app “images helped jog my memory” (M6, 36-49 years), (5) “Using Intake24 is fairly quick” (W10, 18-35 years); and (6) 24HR method preference informed by the final interview question. The final interview question asked participants to summarize their experience of conducting 24HR. Participants expressed wanting the 24HR “… to be as absolutely as easy as possible” (W10, 18-35 years). “I’m just so used to eating something and forgetting about it, and now I was figuring out exactly detail, oriented on what you did and how you ate it and how much you ate” (M3, 50+ years).

### User Experience Content Category 1: Put My Food in the List (M5, 36-49 Years)

For all 24HR methods, participants expressed frustration about trying to find the foods they ate, as food names did not necessarily match what they were searching for. One person suggested that there was no logical naming hierarchy or scaffolding to aid food searching.

It’s like they went for the general names of things rather than that sort of scaffolding the name in a way.

W2, 50+ years

Frustration ensued, despite all food and beverages provided during the feeding study being included in each 24HR food list. For example, one participant did not equate “spinach cannelloni” with “cannelloni veg,” which is how it was named in the IA-24HR food list:

If you are going to give me spinach cannelloni, put it in the (IA-24HR) app [W3, 50+ years)

There were subthemes that described certain factors of the food identification process when comparing the three 24HR methods. Participants felt the food was not in the database, there were too many steps required to search and find the food, and this led to choosing the closest food in their opinion, in the list.

#### My Food Simply Isn’t in the Database (W3, 50+ Years)

Participants described the absence of foods described in their words in the 24HR methods food list as “frustrating,” “time consuming,” and “inconvenient”:

I don’t know if I think about foods differently, but, like there was a lot of like green salad instead of salad greens, or salad coleslaw. You know, it’s kind of like they went for the general names of things rather than like sort of scaffolding the name in a way.

W2, 50+ years

Some felt angry about searching the food lists to find their food and beverage items:

I’m trying to think of a justification why I couldn’t find most of the items I ate, and that’s why it made me quite frustrated. I was like really angry when I was trying to input what I ate.

W4, 50+ years

#### A Few Too Many Steps to Find My Food

Participants said that taking multiple steps to identify their food did not help them recall the types and amounts of foods they had eaten the day before:

When you ask a user to do extra steps, that just makes it harder for the user to complete or wanting to use whatever the method. So try to make it as user friendly, as few steps, as short as few steps required as possible.

W4, 50+ years

#### I Put the Closest Thing I Could Find (W13, 18-35 Years)

Although participants tried to “do the 24HR as correctly as possible” (W13, 18-35 years), “too many steps” led them to choose “the closest thing”:

I guess is it is easier just to tag it as the next best thing, or do you go through the hassle of trying to figure out how to add a new item. And so that was always the annoying bit for all the methods, but you know, I’d always try and do it as correctly as possible.

W10, 18-35 years

### User Experience Content Category 2: It Is Really Hard to Know Portions (M3, 50+ Years)

Estimating portion size was described as the most difficult aspect of using 24HRs, harder than remembering what was eaten, especially for mixed foods such as curry and rice:

I think with all three of them, it’s really hard to know portions and to figure out the different sizes and that kind of stuff, and then to recall how much portions you had by just a picture. So I think that was a pretty difficult way to get your head around it because you’re trying to figure out how much you ate and how much you did and all that kind of stuff

M3, 50+ years

There were three common subthemes that added personal experience detail to the portion estimation step across the three 24HR methods. Only being able to approximate the portion, though some found the life-sized food model booklet in IA-24HR helped, but the perceived lack of a scale in the photos made it hard, despite having the fiducial marker in the image.

#### It Was More of an Approximation (W10, 18-35 Years)

Participants wanted to estimate portion sizes but resorted to “guesstimate” (F7, 36-49 years; M5, 36-49 years), to use “one’s imagination” (M1, 50+ years), and to “approximate” portion sizes (F10, 18-35 years; M1, 50+ years; F3, 50+ years).

…I swear, if it’s like teaspoons and I was like, I don’t know how many teaspoons are in those packs of single serve butter, so I’m going to have to guess at it

W8, 36-49 years

Estimating beverage portions was “very difficult and frustrating” (M12, 18-35 years), and one participant stated “I don’t know how big a cup is” (M11, 18-35 years). The ASA24 [5] and Intake24 [6] show standardized ranges of food portion images to assist portion estimation (Multimedia Appendix 3). Participants felt these comparative portion size images encouraged the “middle” portion or a guess between two images:

When deciding what portion size, because you’re flicking between them, you’re not seeing them all at once. I think I’m talking myself into liking the middle one the most because it’s all there at once, and I’m like oh, that one and you can see it compared to the others. …that’s also a positive because you’re like oh, no a little bit more than that

W10, 18-35 years, ASA24

A total of 10 participants said that they had previously monitored their food intake and that using household measures or food images was frustrating. They wanted to provide more detail, preferring to cite the volume or weight:

“I could probably more accurately tell you the volume or weight of something rather than just the amount, you know, generic content” (M3, 36-49 years) and “Just let me tell you that I’ve drunk 500mLs of water” (M6, 18-35 years).

#### Food Model Booklets Showed the Size (M2, 50+ Years)

The food model booklet (Multimedia Appendix 3) used with the IA-24HR [27] to guide portion estimation assisted some participants:

…because the pictures were relevant to the plates and cups that I’d used

W12, 18-35 years

Most, however, felt the food model booklet hindered their portion size estimation, and the interview with the IA-24HR was unnecessary:

I suppose it did improve my recall of what I did eat, but again, I had trouble with those portion sizes when I was presented with the 2D image.

M13, 18-35 years

#### With a Picture it’s Hard to Know Scale (M3, 50+ Years)

Food portion guides (Multimedia Appendix 3) appear to have confused participants as they tried to estimate portion sizes from the images displayed on the screen (ASA24 and Intake24) or the food model booklet (IA-24HR):

…with a picture, it’s so hard to know scale. So I think that’s probably the main thing, and then trying to remember back, what did you eat? And how much I ate? And that stuff portion guides I remember, it was a pretty stressful time at work to so I was trying to recall things, but I was also focusing on other things as well.

M3, 50+ years

So, taking the photos of the food with that visual comparison, I assumed the plates are of a similar size. Because we were fed on tiny little paper plates and that looks like it could be a standard dinner plate.

W3, 50+ years, IA-24HR

But estimating the size of leftover food was assisted by the mFR images:

The image taken ‘After’ eating in the mFR app helped estimate portions

W4, 50+ years

### User Experience Content Category 3: ASA24 Was a Painful Process (W3:50+yrs)

A total of 13 participants reported that ASA24 was more difficult to complete than the other methods, primarily due to the level of detail requested. Some participants expressed frustration with the process: “Oh God, that was painful, wasn’t it” (W3, 50+ years) and “It was absolutely useless” (W4, 50+ years).

The factors affecting the preference for ASA24 could be summarized into 3 related expressions of their experience. Taking longer than participants expected it to, frustration at the repeated questions, especially for potentially missed foods and beverages, and the language used when technology limited their ability to provide a more exact estimate of how much they ate.

#### ASA24 Took Forever (W3, 50+ Years)

Some participants said the ASA24 “took forever” due to the repetitiveness of the detailed food prompts within the passes, difficulty in finding foods in the database, and the use of portion size shapes with “+” and “–“ buttons:

Yeah, and I know like if it was like tea, then you had to go through tea, and then you had to go through milk, and then you had to go through the portion sizes of everything, it’s just like okay, it’s just a long, long chain.

M3, 50+ years

The ASA24 portion guide tools were challenging and repetitive, even for individual portion items:

I wasn’t a fan of the way ASA24 grouped food items. So, for example, in that image there, you can see you had one egg, and it’s only less than or more than one egg, and then it didn’t ask you exactly the next step, how many eggs you had?

M12,18-35 years

#### I’ve Honestly Said Every Single One This Time I Swear (M6, 36-49 Years)

Participants expressed impatience with the repeated prompts for eating occasions and forgotten foods; “found it was a bit much” (M1, 50+ years). They said continually asking for details made them feel like their honesty was being challenged:

I think I got impatient towards the end because by design, it just kept asking, it kept asking, it kept asking until I said no, there was no more food, I’ve honestly answered. I’ve honestly said every single one this time, I swear.

M6, 36-49 years

Confusion resulted from repeated questioning:

... that was a little bit confusing, I guess I’d say, because you feel like you’ve already put down everything in your meal and then they’re asking you…

W13, 18-34 years

…really made me angry when I was trying to input what I ate... Just don’t recommend this to a user

W4, 50+ years

#### How Many Exactly Did You Have? (M12, 18-35 Years)

Some participants wanted part measures on the ASA24 plus and minus buttons for portion estimation to select from:

I ate one and a half of it, so I don’t think there was an option to actually suggest one and a half. It was either more than one, less than one, and then how many exactly did you have? And it was I ate one and a half, but it was like one or two.

M12, 18-35 years

### User Experience Content Category 4: My Images Helped Jog My Memory (M6, 36-49 Years)

Participants took images before and after eating using the mFR app and labelled their food and beverage images before their IA-24HR interview, during which the images were displayed for them. They thought seeing the images reduced their reliance on memory, improving their ability to remember food and portions:

Photos, they were well lit, easy to see, easy to jog my memory, and go oh that’s right I had that, and I finished it and all that jazz

M6, 36-49 years

Including the novel IA-24HR gave insight into the comparative experience with the established 24HR methods and exposed features that could be further developed. Participants felt they took more notice of what they were eating, found the image helped them remember how much, and the one image showed them what and how much they ate.

#### Took a Bit More Notice Because I Was Taking a Photo. (M11, 18-35 Years)

Taking photos focused participants’ attention on the food eaten, which they felt helped them remember:

So then I was taking the photo, I was actually like looking at what food I was eating, instead of kind of just like, eating stuff

M11, 18-35 years

Taking the photo is probably easier than going back and looking at the actual photo during the interview, that’s me personally that I’d use my memory as a more trusted source I guess

W10, 18-35yrs

Participants expressed that they would have liked to use these images for the other recall methods, but also pointed to a learning effect for repeated 24HRs:

It’s about recall, but by then, I’d learnt if they’re letting you take photos for one of them, then is there anything stopping you writing it down? And I couldn’t see anything that said you can’t write it down. Much like if I’d had the menu in front of me

W3, 50+ years

Remembering to take their food images using the mFR was described as an issue for some:

You just had to remember that you take a picture before you eat it. Because you get so hungry, you’re like, I’m just going to dive in and you’re like, oh, I’ve got to make sure I take a picture before.

M3, 50+ years

#### When You Look at the Photo You Can Remember How Much (W5, 18-34 Years)

The mFR images enabled faster portion estimation for the IA-24HR:

…she did ask the portions of like, the ginger that I had with the sushi; it was easier to remember that I had 8 pieces of ginger because I managed to spread them out evenly on my 8 pieces of sushi. I think it came into my head a lot faster because of the photo, because it would have taken a little bit longer to remember, or I may not have remembered at all—if I didn’t have the photo

W13, 18-34 years

The mFR app images were preferred for recalling food and estimating portions over reference images within the other methods:

I think maybe everyone has a different perception of what they see, so actually, I mentioned before, the method might be the best because you take your own photo, and then when you look at the photo, you can kind of remember how much, what is your intake on that day.

W5, 36-49 years

#### We Can See Clearly What We Chose and What We Ate, That Was Really Helpful (M5, 36-49 Years)

The mFR app images were viewed as helpful in overcoming the challenges of recalling what and how much was eaten during the IA-24HR:

Having the images was a big help because being able to recall exactly and precisely what I ate was a bit more challenging, but having images was the biggest reason why I definitely preferred the app over the other methods.

M12, 18-35 years

Participants agreed that using the mFR app to take food images was “normal” during social interactions. One participant mentioned that others may worry about being judged for their food choices in the images:

…no matter how reassuring a researcher is or something like that, people just feel like they’re being judged, even if they have been reassured many times that they are not.

M6, 36-49 years

### User Experience Content Category 5: Intake24 Is Fairly Quick (M2, 50+ Years)

Participants mentioned that the user interface and software features of Intake24 made it quick:

I just don’t remember it being complicated, it was really quite easy, and it was fairly quick as well. It only took sort of 5 or 10 minutes or so to do ... I think it was just really easy and straightforward.

M2, 50+ years

Intake24 was less repetitive than ASA24 for forgotten foods, but most said they were still able to provide all the information needed:

And it didn’t ask the repeated—have you forgotten anything? And it still had, like, the portioning based on actual images of food, which I liked more than the empty plates and bowls. Not to the detriment to the information provided.

W13, 18-34 years

### User Experience Content Category 6: IA-24HR Recall Method Preference

App and web-based technologies were familiar to participants, and the two self-administered web-based 24HR, ASA24, and Intake24 “were incredibly similar” when viewing a screenshot of the website opening page to assist in recalling the method. More training and support for completing 24HRs was recommended:

You know, other people might enjoy technology but just require a little tutorial in order to be more confident to actually complete it in its entirety.

M13, 18-35 years

Despite each method’s challenges, 8/26 (31%) interviewees specifically mentioned they would use them all again if they needed to, with some participants saying that the task got easier with each new method they tried because they knew what was expected from the study. When asked if they had a preferred 24HR method, using the mFR app to take images was preferred by 20/26 (77%) interviewees, 8 men and 12 women (Multimedia Appendix 7), but some suggested it would be more convenient without the IA-24HR recall interview:

I think the photograph one is the easiest …but again, that was then, finding the time for the interview afterwards.

W10, 18-35 years

## Discussion

### Principal Findings

This study is a unique qualitative investigation of user perceptions of 3 technology-assisted 24HR methods following a randomized crossover controlled feeding study where food and beverage intake was known [12]. Intake24 was found to be the most accurate for energy in the parent study [12], but the user perspective found people preferred to have their own images to estimate the portion sizes and foods eaten. In-depth interviews enabled the comparison of participant perceptions of ASA24, Intake24, and the IA-24HR and provided evidence for future improvements in the 24HR methods. A common experience in conducting the 24-hour recalls was expressed by content analysis result 1 across all methods was “Put my food in the list,” and content result 2 was “It’s really hard to know portions.” Specific features of the methods were summarized in Content 3, 4, and 5. Content 6 states participants wanted the 24HR to be easy to do and showed a preference for using their mFR images collected at the time of eating, as this reduced their reliance on memory when recalling what they ate.

When undertaking self-administered 24HRs, users are required to find their food or beverage using a search function. In this study, both ASA24 and Intake24 were self-administered. Before an interview, participants completing the IA-24HR were asked to label their food images that required finding their food using a search function. With all three 24HR methods, participants had difficulty locating their consumed food and beverages from the descriptions provided, eliciting strong negative emotions. This appeared to be irrespective of the length of the food list (ie, n≥4800 items in ASA24, n≥2800 items in Intake24, and n=372 items in the IA-24HR). The difficulty with finding food names in the food lists is consistent with previous studies assessing ASA24 acceptability among low-income adults [34] and among multiethnic older adults [35]. Difficulty finding foods within the database was also reported with Intake24 [22]. Participants recommended including food naming hierarchies in the mFR app instructions for assisting with image labeling. For example, when searching in the mFR app food list, use a general search term “sushi,” then “salmon sushi” in the second step. This is consistent with suggestions that food naming be user-friendly and designed with both the participants and research end users in mind [36]. The findings in this study indicate that more work is needed to improve the user experience in finding their food.

Similar findings have been reported comparing technology-assisted 24HR methods [21,22]. Although users in the current study were accepting of a self-administered 24HR, some found finding and entering their foods a frustrating task when the exact wording did not match their search. Advances in machine learning [26] and natural language processing have seen an increase in the use of speech data for dietary assessment [37], as well as images used in this study. The integration of such technologies with self-administered 24HR methods warrants further investigation.

When asked to choose a preferred method, participants indicated a preference for using the mFR app to take images of their food at the time of eating, but without the interviewer-administered recall component the following day. Although the reporting time for all three 24HR was less than 30 minutes in the main study [12], participants wanted the process to be even quicker. and that each subsequent recall was easier. The latter is consistent with findings of a learning effect, with repeat recalls requiring less time than those before [38], though the order was not found to affect the accuracy of the recall in the main study [12]. ASA24 and Intake24 are based on an adapted version of the Automated Multiple-Pass Method, which itself was a groundbreaking innovation that has been demonstrated to improve the accuracy of 24-hour recall data [38,39]. Intake24, development included user-centered design during the iterative development to reduce respondent burden with a reduced level of prompting, to which participants in this study responded positively. Collectively, the findings point to the importance of providing participants with support tailored to the specific methods or previous access to the technology to become familiar with the platforms. The provision of advanced access to technology is concluded in a qualitative investigation of Intake24 and ASA24 [Mackenzie 2022]. The ease of use of the 24HR methods was enhanced with the Intake24 video tutorial, more so than the ASA24 self-guided “quick tour.” Technology development testing needs to include the acceptability of methods to the end user, in continued technological innovation to embed advancing technology into dietary research methods beyond the digitizing of traditional methods. Overall, the findings suggest that there is continued room to improve the experience and reduce the burden of completing recalls for participants, with likely implications for study response attrition and data quality [3,15]. As noted above, the findings also point to the need for continued innovation to embed technology in order to advance dietary research methods beyond the digitization of traditional methods. The findings also underscore the need to remain fluid as technology continues to evolve and suggest that there is not likely to be one method that is suitable for all populations, contexts, and purposes.

Estimating portion sizes using the guides within each method was thought to be the most difficult part of the 24HR. 1 With ASA24 and Intake24, participants were presented with a series of static portion size images from which to select. With the IA-24HR method, participants were able to view their food images and were provided with the Australian Health Survey hard copy booklet [27]. Estimating portions using the reference serving plates images was difficult, particularly when the feeding study plate sizes differed from the model displayed. Portion size estimation is a challenging task as it requires a participant to visualize and remember what was eaten using images as a reference point [40]. A systematic review of error in 24HRs and dietary records found that portion misestimation frequently contributed to error [41]. Improving portion size estimation is an opportunity to improve the accuracy of dietary intake data. The current findings suggest some participants may select the middle option of the displayed images or simply guess the amount eaten when estimating portion size. Initial cognitive work underlying the development of ASA24 suggested that accuracy was higher with more versus fewer images and that participants preferred simultaneously rather than sequentially presented images [40]. The original desktop version of ASA24, therefore, presented several images simultaneously, and this has been maintained in Intake24 as thumbnail-sized images. However, with the development of versions for use on small-screen mobile devices, the presentation shifted to show a limited number of images at once, with the opportunity to scroll through images. Although mobile-friendly interfaces expand the possible collection of recalls to numerous settings and contexts, there are tradeoffs between strategies shown to result in the most accurate data and leveraging technology to expand feasibility and improve the respondent experience [12,42]. Incorporating qualitative studies, such as the current investigation comparing the new IA-mFR to established digitized versions of traditional methods, ASA24 and Intake 24, considers the user’s perspective in method development. This approach is novel as reviews of electronic health interventions found diverse evaluation methods have been used in the development of novel digital health technology, which are often not compared to traditional methods [43,44].

The preference for taking images to assist in the recall process was a key finding of this study. Access to their mFR images during recall facilitated ease of use and reduced reliance on memory. In this study, participants labelled the content of their food images, identifying the foods linked to a food composition database. The time and date were extracted from the image metadata, thereby reducing the burden on participants to report this data. Iterations of the mFR app may shift the confirmation of the content of the images from the participant to automated methods using computer vision and machine learning techniques under development [19,25,26,45]. Advances in machine learning techniques for food identification and portion size estimation are needed and will likely further reduce participant burden for these tasks.

The in-depth interviews elicited an understanding of the personal context for completing the 24HDR, such as at home or work, or any expectations from previous experience. Participants in this study reported that work-related stresses affected the time available to complete the 24HR. This is consistent with a think-aloud study [21], in which some participants who pressed for time experienced frustration or anger and thought the 24HR was excessively demanding. Study participants described how using the mFR app images reduced memory burden and how the ASA24 and Intake24 web interfaces enabled them to conduct their recalls independently and privately on their own devices. It is possible that the preference for not participating in the IA-24HR interview using video conferencing may have been influenced by time and work stress factors, as most participants were in full-time employment. The results from the controlled feeding study found that a trained analyst was able to estimate portion size for energy and macronutrients from images more accurately than with the IA-24HR participant interview method [12]. Recent work [45] on advancing automated portion estimation from images could address some of the participant burden experienced in this study.

### Strengths and Limitations

This is the first study, to our knowledge, to use inductive content analysis to compare user experiences related to using three different 24HRs (ASA24, Intake24, and IA-24HR) undertaken with a controlled crossover feeding study methodology. The semistructured interview enabled a rich discussion of the participants’ experience of the phenomenon of 24HR. While we recruited 26 interviewees (17% of ACE-TADA participants), there may have been perspectives that were missed from inclusion in the main study*.* The 24HR methods assigned at the first meal at the start of the feeding study were allocated in a randomized order to reduce a training effect bias during the subsequent 24HR methods. The order of the 24HR methods was evenly distributed for the current qualitative study, and in the ACE-TADA larger study, it was not associated with the accuracy of 24HR estimation of energy intake [12]. Potential limitations include unavoidable training from experiencing three 24HRs. Additionally, the participants were recruited from the university community, which has a high level of educational attainment and digital literacy, potentially increasing their acceptance of the technology-based features. However, for some, this did not reduce their frustration with finding their food in the food lists.

The generalizability of these results is limited by the main study participants from whom this sample was recruited. The study participants for the ACE-TADA study were recruited from staff and students at a university facility. The original protocol had planned to recruit from the electoral roll, a compulsory enrolment system for Australians aged 18 years and older, but COVID-19-mandated restrictions only allowed staff and students on campus at the time. Therefore, the sample selected is unlikely to be representative of the general population in Australia. The sample had higher education levels, was younger, with a higher proportion of Asian individuals than in the general population [12]. Interpretation of the findings in this study should consider the limitations of this sample. For example, the preferences for image-assisted methods may reflect the younger demographic we recruited. Future work should include people with a greater diversity of ethnicity and socioeconomic status to confirm if the user experience and preferences observed in this study are replicated.

### Conclusion

User experience of preferences when comparing three technology-assisted 24HR methods highlights the burdensome and frustrating challenges for users related to finding foods and estimating portion sizes, and preferences for wanting 24HRs to be as easy as possible. Participants’ preferred method was the IA-24HR with access to their mFR images taken at the time of eating. Findings highlight the importance of using qualitative methods to explore user preferences for dietary assessment methods and confirm the need to reduce the respondent user burden associated with 24HR methods. These results point to the next steps in developing 24HR dietary assessment methods, which would be to focus on improving the user experience. The results of this study indicate user frustration with the food identification and portion size estimation. In the future, automating food identification and portion size estimation with machine learning and artificial intelligence may lead to an enhanced user experience.

## Appendix

Multimedia Appendix 1 Feeding study sample menu for three meals in a day.

Multimedia Appendix 2 Characteristics of the three technology-assisted 24-h dietary recalls (ASA24a, Intake24, and IA-24HRb) assessed with in-depth interviews, showing the features of each method.

Multimedia Appendix 3 Portion estimation characteristics of the three technology-assisted 24-h dietary recalls: A: ASA24, B: Intake24, and C: IA-24HR showing the food model booklet image for the estimation of portion sizes by participants.

Multimedia Appendix 4 ACE-TADA (Accuracy and Cost-Effectiveness of Technology-assisted Dietary Assessment) exit interview script to understand the comparative user experience of three 24HR (24-hour dietary recall) methods.

Multimedia Appendix 5 COREQ reporting checklist for qualitative studies.

Multimedia Appendix 6 Demographic characteristics of interview participants (n=25) who had completed three 24HR (24-hour dietary recall) methods.

Multimedia Appendix 7 Preferred 24HR (24-hour dietary recall) method.

## Abbreviations

24HR: 24-hour dietary recall
ACE-TADA: Accuracy and Cost-effectiveness of Technology-Assisted Dietary Assessment
AMPM: Automated Multiple-Pass Method
ASA24: Automated Self-Administered Dietary Assessment Tool-2016
AUSNUT: Australian Food and Nutrient Database
COREQ: Consolidated Criteria for Reporting Qualitative Research
IA-24HR: Image-Assisted Interviewer-Administered 24-hour dietary recall
mFR: mobile Food Record
SCRAN24: Self-Completed Recall and Analysis of Nutrition

## Data Availability

The datasets generated and analyzed during this study are available from the corresponding author on reasonable request.
