# Supplementary material for: User Preferences for an Image-Assisted Dietary Recall: Qualitative Study Comparing 3 Dietary Assessment Methods
Source: JMIR Hum Factors. 2025 Dec 30;12:e79565. doi: 10.2196/79565 (PMC12811038; doi:10.2196/79565)
Supplement: Multimedia Appendix 1 [file humanfactors_v12i1e79565_app1.pdf]

ACE TADA study

NAME: __________ Date: 16/12/2021

Researcher ID: ____ Time: __

DAY THREE BREAKFAST MENU

BREAKFAST

- ### CEREAL

Cornflakes, Weet-bix, Special K, Natural muesli, Porridge with your choice of milk

Optional extras: Strawberries, Banana**,** Peaches (tinned) Greek yoghurt and fruit yogurt , honey, brown sugar.

- ### TOAST

Choose between Multigrain, White, Ciabatta and Fruit bread

Spread Options: Jam, Vegemite, Honey, Peanut paste, Marmalade, Butter, Margarine, Avocado with a slice of lemon

EGGS– Poached hard or soft? let us know how you like them.

GRILLED TOMATO AND MUSHROOMS– sliced tomato and whole button mushrooms grilled and comes with a balsamic dressing on the side.

CAPPUCCINO MUFFIN: Homemade coffee choc chip muffin

DRINKS

HOT DRINKS

- **TEA–** English breakfast, Earl Grey, Green, Peppermint, Ginger and lemon
- **Coffee**– instant , espresso or flat white

OPTIONS

Milk– full cream, hilo or skim

Soy milk -light and regular), almond milk, lactose-free

COLD DRINKS

- **WATER**– tap, sparkling
- **JUICES** -Apple , Orange

EATING RESEARCH PERTH

ACE TADA study

NAME: ____________ Date: 16/12/2021

DAY THREE BREAKFAST MENU

Researcher mFR_______TIME:___________

Indicate your menu choices by circling your request and writing how many you would like where appropriate.

	BREAKFAST MEAL	Office use only
**CEREAL**	Cornflakes, Weet-bix, Special K Nat Muesli Porridge	
MILK	Regular HiLo Skim Soy Soy Lite Almond Lactose-free	
YOGHURT	Greek Natural yoghurt Fruit flavoured Yoghurt	
FRUIT	Strawberry Banana Peaches Brown sugar Honey	
**TOAST**	Fruit bread Ciabatta Multigrain White (#)	
SPREADS	Jam Marmalade Honey Peanut paste Vegemite (#)	
	Margarine Butter Avocado Lemon	
**HOT**	Eggs– poached soft OR hard	
	Grilled Tomato Grilled Mushroom Balsamic dressing	
	Cappuccino muffin	

	BREAKFAST DRINKS	Office use only
**WATER**	Water Still Sparkling Water	
**JUICE**	Orange Apple	
**TEA**	with milk OR without milk Earl Grey Eng Bfast Green P'mint	
**COFFEE**	with milk OR without milk Instant Espresso Flat white	
**MILK**	Soy Soy lite Full cream HiLo Skim Milk Almond Lactose-free	
	**Sweetener or Sugar (number)**	

EATING RESEARCH PERTH

ACE TADA study

NAME: __________Date: 16/12/2021

Researcher ID: ________TIME:_________

DAY THREE MENU

LUNCH

SUSHI:

- Vegetarian
- Chicken
- Smoked Salmon

RICE PAPER ROLLS.

- Chicken and veg
- Tofu and veg

Optional extras: wasabi, soy sauce and pickled ginger

WRAPS: Fresh salad wrap.

- **Options:** chicken, ham or avocado each available with cheese .

TOASTED SANDWICH:

- Cheese and ham and tomato

SALAD:

- Green salad– lettuce, tomato and cucumber with a side of dressing

SOMETHING TO FINISH

Fruit salad, Chocolate brownie home made

DRINKS

HOT DRINKS

- **TEA–** English breakfast, Earl Grey, Green, Peppermint, Ginger and lemon
- **Coffee**- instant , espresso or flat white

OPTIONS

Hilo milk, full cream, soy milk (light and regular) , skim milk, almond milk, lactose-free

COLD DRINKS

- **WATER**- tap, sparkling
- **JUICES -**Apple , Orange
- **SOFT DRINKS** Diet coke, Coke, Coke zero, Ginger beer, Lemon squash, Lemonade, Lemonade diet

EATING RESEARCH PERTH

ACE TADA study

NAME: __________ Date: 16/12/2021

DAY THREE LUNCH MENU

Researcher ID__________ TIME:_____________

Indicate your menu choices by circling your request

After a small portion? Tick this box ☐

LUNCH MEAL		Office use only	Extras	√	Office use only
SUSHI	Vegetarian sushi slice (#)				
	Chicken sushi slice (#)		Pickled ginger		
	Smoked salmon sushi slice (#)		Wasabi		
RICE PAPER ROLLS	Chicken rice paper rolls (#)		Soy sauce		
	Tofu rice paper rolls (#)				
WRAP	Chicken OR Ham Or Avocado Cheese				
SALAD	Green salad Dressing				
TOASTED	Combined OR Cheese Ham Tomato				
DESSERT	Chocolate brownie Fruit salad				

LUNCH DRINKS		Office use only
WATER	Water still Sparkling water	
JUICE	Apple Orange juice	
SOFT-DRINK	Coke Coke Zero Diet Coke	
	Ginger beer Lemonade Lemonade diet Lemon squash	
TEA	with milk OR no milk Earl Grey Eng Bfast Green P'mint	
COFFEE	with milk OR no milk Instant Espresso Flat white	
MILK	Soy Soy lite HiLo Skim Full Cream Almond Lactose-free	
	**Sweetener or Sugar (number)**	

EATING RESEARCH PERTH

ACE TADA study

NAME: ____________Date: 16/12/2021

DAY THREE DINNER MENU

Researcher ID: ______ __TIME:__________

DINNER

- ## MAIN MEALS

CURRY:

- Thai green chicken curry—spicy

PASTA:

- Beef lasagne
- Spinach and ricotta tortellini

STIR FRY: Teriyaki chicken noodle and vegan rice

ROAST MEAT : Slices of roasted chicken or beef with gravy. These can also be added to any item.

VEGETABLE OPTIONS :

- Greek style salad—cucumber, tomato, olive, fetta cheese with dressing
- Steamed mixed vegetable- broccoli, carrots and corn
- Baked potatoes in herbed butter

SOMETHING TO FINISH

Fruit salad, chocolate brownie and individual ice cream –Drumstick or Magnum.

DRINKS

COLD DRINKS

- **WATER**- still or sparkling
- **JUICES** -Apple or Orange
- **SOFT DRINKS** Diet coke, Coke, Coke zero, Ginger beer, Lemon squash, Lemonade, Lemonade diet

EATING RESEARCH PERTH

ACE TADA study

NAME: ____________ Date: 16/12/2021:

DAY THREE DINNER MENU

Researcher mFR_______ TIME:__________

Indicate your menu choices by circling your request

After a small portion? Tick this box ☐

	DINNER MEAL	Office use only
**Curry**	Thai green chicken (spicy)	
**Pasta**	Beef lasagne Spinach and ricotta tortellini	
**Stir fry**	Chicken teriyaki OR vegan rice	
**Roast**	Chicken Beef slices Gravy	
**Vegetables**	Greek style salad Steamed veg Baked potato	
**Dessert**	Fruit salad Brownie Drumstick Magnum	

	**DINNER DRINKS**	Office use only
**WATER**	Water still Sparkling water	
**JUICE**	Apple Orange juice	
**SOFTDRINK**	Coke Diet coke, Coke zero,	
	Ginger beer Lemonade Lemonade diet Lemon squash	

EATING RESEARCH PERTH
