# Supplementary material for: User Preferences for an Image-Assisted Dietary Recall: Qualitative Study Comparing 3 Dietary Assessment Methods
Source: JMIR Hum Factors. 2025 Dec 30;12:e79565. doi: 10.2196/79565 (PMC12811038; doi:10.2196/79565)
Supplement: Multimedia Appendix 2 [file humanfactors_v12i1e79565_app2.docx]

**Table S1.** Characteristics of the three technology-assisted 24-h dietary recalls (ASA24^a^, Intake24, and IA-24HR^b^) assessed with in-depth interviews, showing the features of each method.

|  | ASA24 | Intake24 | IA-24HR^b^ |
| --- | --- | --- | --- |
| **Food composition database** | > 4,800 foods/beverages from the Australian food nutrient database | > 2,800 foods/beverages from the Australian food nutrient database | 372 foods/beverages from the Australian food nutrient database |
| **Administration** | Self-administered | Self-administered | Interview-assisted |
| **Interface** | Website | Website | mFR^c^ app and online interview |
| **Time and occasion** | Recall prompts with 8 meal labels and time | Recall prompts are named eating occasions: meals, snacks, beverages and to self-select time | Time of eating automatically captured from image metadata |
| **Food identification** | Selected from searchable food list after recording time of eating | Selected in 2 steps:  from a short list when recording eating add detail from a searchable food list | At time of eating, users record an image of their meal using the mFR app.  After midnight images available for participant labelling |
| **Portion size estimation** | Standard  food/beverage images  assisted portion size estimation. Recall by selecting from portion image, with greater (+) or less (-) symbols to select portion size | Standard food/  beverage images  assisted portion size estimation. Recall by selecting from a range of portion images to indicate size | Participants viewed their mFR images to assist portion estimation and the food model booklet to identify amounts |
| **Forgotten foods** | Automated prompts after final review for commonly forgotten foods with ‘yes’ or ‘no’ forced answers | Automated prompts for items typically consumed together and beverages | Interviewer-administered forgotten images pass |

^a^ASA24 Automated Self-Administered Dietary Assessment Tool-2016, ^b^ IA-24HR- Image assisted interview administered 24 hour dietary recall, ^c^mobile food record.
