# Supplementary figures and images for: User Preferences for an Image-Assisted Dietary Recall: Qualitative Study Comparing 3 Dietary Assessment Methods
Source: JMIR Hum Factors. 2025 Dec 30;12:e79565. doi: 10.2196/79565 (PMC12811038; doi:10.2196/79565)

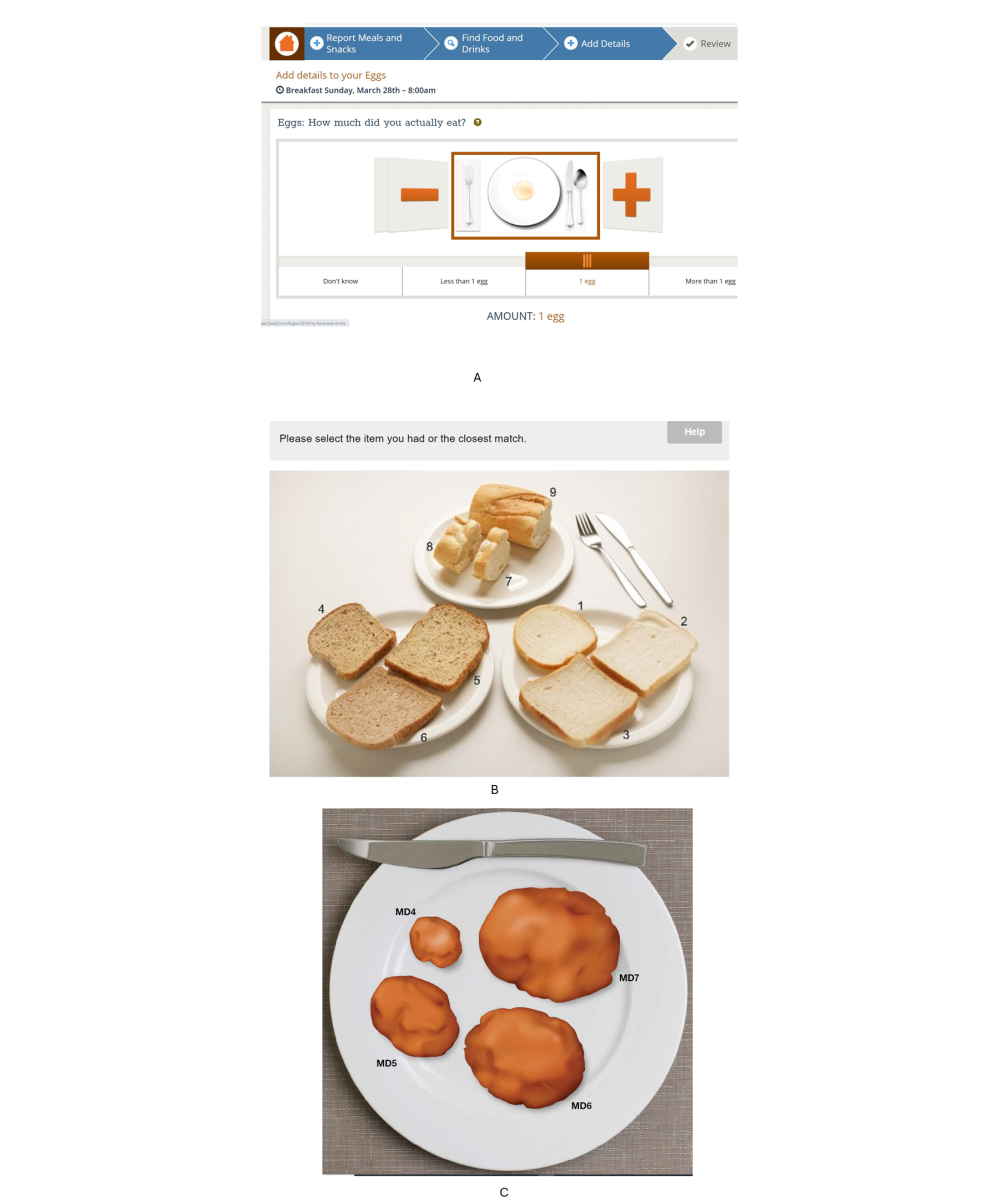

Supplement: Multimedia Appendix 3 [file humanfactors_v12i1e79565_app3.png]
