# Supplementary material for: User Preferences for an Image-Assisted Dietary Recall: Qualitative Study Comparing 3 Dietary Assessment Methods
Source: JMIR Hum Factors. 2025 Dec 30;12:e79565. doi: 10.2196/79565 (PMC12811038; doi:10.2196/79565)
Supplement: Multimedia Appendix 4 [file humanfactors_v12i1e79565_app4.docx]

# ACE TADA exit interview script

Overall Aim:

1. To explore the acceptability, preference and perceptions of three technology-assisted 24 hour dietary recalls (24HR) using in depth interviews

## Participant selection:

- 15-20 participants selected from those completing the ACE-TADA controlled feeding study who have undertaken all three 24HR dietary recall methods: ASA24, Intake24, image-assisted mobile food record 24HR (mFR24) and who have indicated a willingness to undertake an interview.
- Approximately equal number of men and women
- Distribution across age groups: 18-35yrs, 35-50 yrs, 50 + yrs.
- NVivo used to organise codes and themes.

## Introduction:

I am from The Eating Research team at Curtin University where you signed up for the eating study a few months ago. Thank you for taking the time to chat with me today. I would like to ask you some questions over the next 30-45 minutes about your experiences in the study.

With your permission, I would like to record what you say. The audio recording is to capture the feedback you are providing. The recording will only be accessible to program staff and you will not be identified in any publications or reports from this study. You will be able to review the written transcript before any analysis is started (Phone recording turned on with permission).

So, what I would like you to do now is ask you to remember the three feeding days in the food laboratory. Each time, on the following day, you were asked to recall everything you ate or drank in the previous 24 hours. Each week, you tested a different recall method to recall what you ate and drank the day before. For two methods, you completed a survey online, known as ASA24 and Intake24. For the third method, you took photos of your meals and snacks using the mFR app and attended an online interview the next day. At the interview, you were able to view the photos you took in the app and a food model book to help recall what you had eaten. (**share screen shots of the three methods Appendix 1**)

*Does this sound familiar to you? Is there anything you would like to clarify before we continue?*

**A few important points to remember are:**

- There are no right and wrong answers – we would just like to know what *you* think.
- You do not have to answer any questions that make you feel uncomfortable or would prefer not to answer.
- If anything is not clear or you have anything further to add - please stop me at any time.

I would like to start by talking about your overall experiences with the study

- Could you tell me what you thought about the study in general?
- Was there anything you were hoping to get out of the study?

**The following questions are about your experiences and opinions of the three different recall methods you undertook.**

*[order of discussion to be randomised according to their block randomisation]*

Just for some background, the 24-hour recall method is used in our Australian Health Survey to help inform governments and health professionals about what Australian’s are eating. This method is also used by nutrition researchers.

When researchers have talked to groups of people just like you about the 24HR method, some people told us that they liked doing the recall, remembering what they ate the day before and others were not so sure. And so what I am looking for today is **what you think about these methods and any ideas you may have to make things easier** because you’re the expert.

So we really want to learn about what you have to say, so that’s why we’re going to have a chat today about what parts of each recall method you found:

- helpful or
- not so helpful and
- suggestions for how they could be improved.

**We will talk about them in the same order that you completed them.**

- **mFR**

**The following questions are about your experiences and opinions of the mFR app and interview recall method** (Capturing barriers, enablers and incentives to participation)

For this method, you downloaded the mFR App to your phone and recorded images before and after your meal using the app. You identified the food items from a list and used the images to help with your recall interview the next day. You were also provided with a food model book to help with estimating how much you ate.

- Could you tell me what you thought about the:
  - mFR24?
- Was there anything you would like to tell me about your experience with the app?
  - (Further prompts: enjoyed it, practical, easy to move between screens, interface was easy to learn, I could see when the images had uploaded or the pins confirmed )
  - Could you describe how you felt about taking your food images?

(eg affect social interactions?)

- Thinking about the next day where you were asked to label your food images, can you tell me a bit more about how you found using the app to label your food images?
  - - How do you think others (e.g. friends, family) would go with this?
    - Can you think of what might make this easier?
- Thinking about the interview now, was there anything you would like to tell me about your experience?
  - How did you find using your own images for the recall?
  - How did you find using the food model booklet to help with recalling amounts?
    - Can you tell me a little more about that?
  - Were there any parts of the mFR recall/interview that you found more challenging?
    - Can you tell me a little more about that?
- Thinking about where you were when you completed the mFR interview, can you tell me how you felt about actually doing the recall via teams/Zoom?
  - ( eg: time of day and time taken to complete- was this expected, longer or shorter)

Can you think of anything else that we could have provided that might have helped you complete the recall? (e.g. having feedback on your images?)

**ASA24 recall method** (Capturing barriers, enablers and incentives to participation)

Screen shot of ASA24 shown

This was one of the online recalls you completed independently, by yourself, on your own. You were guided through questions on food and beverages consumed at each meal, with images in the website to guide portion size estimation and the recall included a meal-based quick list, detail pass, final review and forgotten foods review.

- Could you tell me what you thought about:
  - ASA24?
- Was there anything you would like to tell me about the website?
  - (eg enjoyed it, practical, easy to move between screens)
- Were there any parts of the recall that were easier to complete?
  - Can you tell me a little more about that?
- Were there any parts of the recall that you found more challenging?
  - ( eg How did you complete the record; recipes or single ingredients)
  - Can you tell me a little more about that?
- Thinking about where you were when you completed the recall, can you tell me how you felt about actually doing the recall?
  - ( eg: time of day and time taken to complete- was this expected, longer or shorter )
- Can you think of anything else that we could have provided that might have helped you complete the record?

**The following questions are about your experiences and opinions of the Intake24 recall method** (Capturing barriers, enablers and incentives to participation)

Screen shot of Intake24 shown

This was an online recall you completed on your own with images in the website to assist in estimating the portion sizes for some of your foods and drinks.

- Could you tell me what you thought about:
  - Intake24?
- Was there anything you would like to tell me about the website?
  - (eg enjoyed it, practical, easy to move between screens)
- Were there any parts of the recall that were easier to complete?
  - Can you tell me a little more about that?
- Were there any parts of the recall that you found more challenging?
  - ( eg How did you complete the record; recipes or single ingredients)
  - Can you tell me a little more about that?
- Thinking about where you were when you completed the recall, can you tell me how you felt about actually doing the recall?
  - ( eg: time of day and time taken to complete- was this expected, longer or shorter )
- Can you think of anything else that we could have provided that might have helped you complete the record?

**Based on the experience you have had with the three dietary recall methods, can you tell me if you were to undertake a recall again, which of the three methods if any, you would prefer?**

- You have selected [ASA24 or Intake24 or mFR24] can you say a bit more about your preference for this method? [prompt: what they liked/disliked compared to the other methods].
- [if said no preference] any reasons as to why no preference?
- Thinking about your friends and family, is there one method that you feel others may prefer?
  - Can you explain that a bit more?

**That’s the end of the interview. I will briefly review what we have covered today. A bit like a forgotten foods recap, so please stop me to add or clarify any points where you feel the need to. I wanted to let you know that it’s what you actually say that’s really important.**

Provide a brief recap of points raised though clarification of concepts will occur throughout the interview. We have a $50 voucher for Coles, Myer or Bunnings (you can choose) to thank you for your valuable time.
