# Supplementary material for: User Preferences for an Image-Assisted Dietary Recall: Qualitative Study Comparing 3 Dietary Assessment Methods
Source: JMIR Hum Factors. 2025 Dec 30;12:e79565. doi: 10.2196/79565 (PMC12811038; doi:10.2196/79565)
Supplement: Multimedia Appendix 5 [file humanfactors_v12i1e79565_app5.docx]

Multimedia Appendix 5: Table 2 COREQ^*^ checklist for Feeding study - exit interviews.

|  |  |  |  |  |
| --- | --- | --- | --- | --- |
| **Domain 1: Research team and reflexivity** | |  | Response |  |
| Personal characteristics | | |  |  |
|  | Interviewer/facilitator | Which authors conducted the interview or focus groups | JDH interview, transcript review and writing of draft manuscript. | Pg 4 |
|  | Credentials | What were the researchers’ credentials | Title page has all the credentials of the researchers | Title page |
|  | Occupation | What was their occupation at the time of the study | JDH- APD (dietitian) PhD candidate, research assistant  AH APD, research assistant | Title page and Pg 4 |
|  | Gender | Was the researcher male or female | All women | Pg 5 |
|  | Experience or training | What experience or training did the researchers have | JDH: APD (dietitian) with mid-career level practice with community-dwelling clients in private practice and CVD rehab, transcript coding and note-taking during focus group workshops.  AH: APD early career practice, online training in NVivo, research in content analysis.  CMP: APD established dietetic, public health and research career, facilitated focus groups, workshops,. | Pg 5 |
| Relationship with participants | | |  |  |
|  | Relationship established | was a relationship established prior to study commencements | JDH had contact with some interview participants in the conduct of the feeding study. JDH prepared the ordered meals with incidental contact with ACE-TADA participants. | Pg 4 |
|  | Participant | what did the participants know about the researchers- personal goals and reasons for doing the research | A general introduction explaining the reasons for doing the research were stated before the interview started. Explicit personal goals for conducting the research were not expressed by the researcher. | Pg 4 |
|  | Participant knowledge of the interviewer | what characteristics were reported about the interviewer/facilitator e.g. bias, assumptions, reasons and interested in the research topic. | Explicit personal goals for conducting the research were not expressed by the researcher | N/A |
|  | Interviewer characteristics | what characteristics were reported about the interviewer/facilitator eg bias, assumptions, reasons and interested in the research topic. |  | Pg 4 |
| **Domain 2; study design** | | |  |  |
| Theoretical framework | |  |  |  |
|  | Methodological orientation and theory | what methodological orientation was stated to underpin the study? Grounded theory, discourse analysis, ethnography, phenomenology, content analysis | Phenomenology as we were interested in their firsthand perceptions and attitudes to using the recall methods and motivations to participate in dietary monitoring studies. | Pg 4 |
| Participant selection | | |  |  |
|  | Sampling | How were participants selected eg purposefully, convenience, consecutive, snowball | Participants were selected from the ACETADA feeding study who had completed three 24HDR methods and the final preference survey. They were purposefully sampled to ensure sex and age groups were represented. | Pg 5 |
|  | Method of approach | How were participants approached- email, f2f, telephone, mail | Participants who had completed all three 24HR methods were purposefully invited by email to be interviewed | Pg 4 |
|  | Sample size | How many participants were in the study | 26 participants completed the interview | Pg 4 |
|  | non-participation | How many people refused to participate or dropped out? Reasons? | A total of 35 participants were invited (16 male and 19 female) and follow up emails were sent after 1 week if there was no response. Two participants did not continue after booking an interview and no reason was sought for no response to the email invitations. | Pg 4 |
| Setting |  |  |  | |
|  | Setting of data collection | where was the data collected eg home, clinic, workplace | The interviews were conducted online though the timing for some interviews indicated that the participants were either at work or at home. | Pg 4 |
|  | Presence of non-participants | was anyone else present besides the participants and researchers | In the online interview there was no-one else included. | Pg 4 |
|  | Description of sample | what are the important characteristics of the samples? Demographics data, date, place | Half the (n=26) interviewees were women (n=13), mean age 40.5±9.2 years (32% <35 years, 40% 35 to 49 years, and 28% 50 years or older), and 54% (n=14) of white ethnicity. | Pg 6 |
| Data collection | |  |  |  |
|  | Interview guide | Were questions, prompts, guides provided by the authors? Was it pilot tested? | The interview script was developed by the research team experienced in dietary assessment and qualitative methods (JDH, CMP, DAK). | Pg 5 |
|  | Repeat interviews | Were repeat interviews carried out? If yes, how many? | No repeat interviews were conducted. | N/A |
|  | Audio/video recording | did the researcher use recording to collect the data | Audio recordings | Pg 5 |
|  | Field notes | were field notes made during and/or after the interview or focus group | The interviewer made brief notes whilst conducting the interview to assist with prompting further exploration of points raised by the interviewee. These were not used in any analysis conducted | N/A |
|  | Duration | What was the duration of the interviews or focus groups | The interviews took an average of 31.4 minutes (range 23-42.25 minutes) | Pg 6 |
|  | Data saturations | was data saturation discussed? | Recruitment of interviewees continued until pragmatic saturation, where no new points were stated in the interviews | Pg 6 |
|  | Transcripts returned | were transcripts returned to participants for comment and/or correction | Participants were invited to review them for accuracy; five of the 26 reviewed their transcripts with two edits | Pg 5 |
| **Domain 3: analysis and findings** | | |  |  |
| Data analysis | |  |  |  |
|  | number of data coders | how many data coders coded the data | Three women coders (JDH; AH; CMP) with diverse backgrounds and research experience, independently reviewed the transcripts | Pg 5 |
|  | Description of the coding tree | did authors provide a description of the coding tree | The analysis was conducted iteratively so there was not an *a priory* coding tree | N/A |
|  | Derivation of themes | were themes identified in advance or derived from the data | Themes were derived from the data | Pg 5 |
|  | Software | what software, if applicable, was used to manage the data? | NVivo software was used for the data analysis | Pg 5 |
|  | Participant checking | did participants provide feedback on the findings | No | N/A |
| Reporting | |  |  |  |
|  | quotations presented | were participant quotations presented to illustrate themes/findings | Yes, description text of themes and sub themes | Results section |
|  |  | was each quotation identified e.g. participant # | Quotes are identified by a number, sex and age of the participants is shown to demonstrate the diversity of the responses. | Results section |
|  | data and findings consistent | there was consistency between the data presented and the findings | Yes | Results section |
|  | clarity of major themes | were major themes clearly presented in the findings | Yes | Results section |
|  | clarity of minor themes | is there a description of diverse cases or discussion of minor themes | Yes | Results section |

* Harris JE, Gleason PM, Sheean PM, Boushey C, Beto JA, Bruemmer B. An Introduction to Qualitative Research for Food and Nutrition Professionals. Journal of the American Dietetic Association. 2009;109(1):80-90. doi:10.1016/j.jada.2008.10.018
