# Supplementary material for: User Preferences for an Image-Assisted Dietary Recall: Qualitative Study Comparing 3 Dietary Assessment Methods
Source: JMIR Hum Factors. 2025 Dec 30;12:e79565. doi: 10.2196/79565 (PMC12811038; doi:10.2196/79565)
Supplement: Multimedia Appendix 6 [file humanfactors_v12i1e79565_app6.docx]

## Multimedia Appendix 6

Table 3: Demographic characteristics of Interview participants

| Characteristic | | Exit interview n=26  n (%) |
| --- | --- | --- |
| Gender | Female | 13 (50) |
|  | Male | 13 (50) |
| Age | < 34 yrs | 8 (32) |
|  | 35-49 yrs | 10 (40) |
|  | 50+ yrs | 7 (28) |
| BMI visit 1 (kg/m2), mean (SD) |  | 26.5 (4.4) |
|  | Female | 25.9 (5) |
|  | Male | 27.2 (4) |
| Ethnicity | Asian | 11 (42) |
|  | Caucasian | 14 (54) |
|  | Aboriginal | 1 (4) |
| Education level achieved | Bachelor degree or higher | 24 (92) |
|  | Year 10 or 12 equivalent | 2 (8) |
| Employment status | Full time | 20 (77) |
|  | Part-time/casual | 4 (15) |
|  | Student | 2 (8) |
