# Supplementary material for: User Preferences for an Image-Assisted Dietary Recall: Qualitative Study Comparing 3 Dietary Assessment Methods
Source: JMIR Hum Factors. 2025 Dec 30;12:e79565. doi: 10.2196/79565 (PMC12811038; doi:10.2196/79565)
Supplement: Multimedia Appendix 7 [file humanfactors_v12i1e79565_app7.docx]

## Multimedia Appendix 7

Table 4: Preferred method of 24HR stated by interviewees after participating in a three-arm crossover study comparing three 24HR methods.

| 24HR Method |  | Men (n=13) | Women (n=13) |
| --- | --- | --- | --- |
|  |  | n (%) | n (%) |
| IA-24HR |  |  |  |
|  | 18-34 years | 3 (23) | 3 (23) |
|  | 35-49 years | 4 (31) | 5 (38) |
|  | 50+ years | 1 (8) | 4 (31) |
| Intake24 |  |  |  |
|  | 18-34 years | 1 (8) | 1 (8) |
|  | 35-49 years | 1 (8) |  |
|  | 50+ years | 1 (8) |  |
| Either web-based | 50+ years | 1 (8) |  |
| No preference | 35-46 years | 1 (8) |  |
| IA-24HR- Image-assisted Interview administered 24 hour dietary recall | | | |
